# PHi-C2: interpreting Hi-C data as the dynamic 3D genome state

**DOI:** 10.1093/bioinformatics/btac613

**Published:** 2022-09-10

**Authors:** Soya Shinkai, Hiroya Itoga, Koji Kyoda, Shuichi Onami

**Affiliations:** Laboratory for Developmental Dynamics, RIKEN Center for Biosystems Dynamics Research, Kobe 650-0047, Japan; Laboratory for Developmental Dynamics, RIKEN Center for Biosystems Dynamics Research, Kobe 650-0047, Japan; Laboratory for Developmental Dynamics, RIKEN Center for Biosystems Dynamics Research, Kobe 650-0047, Japan; Laboratory for Developmental Dynamics, RIKEN Center for Biosystems Dynamics Research, Kobe 650-0047, Japan; Life Science Data Sharing Unit, Infrastructure Research and Development Division, RIKEN Information R&D and Strategy Headquarters, Kobe 650-0047, Japan

## Abstract

**Summary:**

High-throughput chromosome conformation capture (Hi-C) is a widely used assay for studying the three-dimensional (3D) genome organization across the whole genome. Here, we present PHi-C2, a Python package supported by mathematical and biophysical polymer modeling that converts input Hi-C matrix data into the polymer model’s dynamics, structural conformations and rheological features. The updated optimization algorithm for regenerating a highly similar Hi-C matrix provides a fast and accurate optimal solution compared to the previous version by eliminating the factors underlying the inefficiency of the optimization algorithm in the iterative optimization process. In addition, we have enabled a Google Colab workflow to run the algorithm, wherein users can easily change the parameters and check the results in the notebook. Overall, PHi-C2 represents a valuable tool for mining the dynamic 3D genome state embedded in Hi-C data.

**Availability and implementation:**

PHi-C2 as the phic Python package is freely available under the GPL license and can be installed from the Python package index. The source code is available from GitHub at https://github.com/soyashinkai/PHi-C2. Moreover, users do not have to prepare a Python environment because PHi-C2 can run on Google Colab (https://bit.ly/3rlptGI).

**Supplementary information:**

Supplementary data are available at *Bioinformatics* online.

## 1 Introduction

High-throughput chromosome conformation capture (Hi-C) quantifies genomic DNA contacts in the three-dimensional (3D) conformation of chromosomes across the whole genome (Lieberman-Aiden *et al.*, 2009). The processed data are typically combined into a matrix as the population-averaged contact probability, which is depicted as a two-dimensional (2D) heatmap (Kerpedjiev *et al.*, 2018; Robinson *et al.*, 2018). The various 2D Hi-C patterns should reflect the structural characteristics of the 3D genome organization. However, since Hi-C data consist of a mass of snapshots of proximal genomic DNA pairs due to chemical fixation, the outcome pictures are mostly limited to static and averaged models. Meanwhile, live-cell imaging has revealed that chromatin dynamically moves, coupling with genome functions within living cells (Heun *et al.*, 2001; Nagashima *et al.*, 2019). Therefore, biophysical modeling is essential for developing a quantitative understanding of the gap between Hi-C data for fixed cells and information on chromatin dynamics for living cells.

In 2020, we released PHi-C software as Python codes designed to decipher Hi-C data into polymer dynamics (Shinkai *et al.*, 2020b). PHi-C demonstrations output dynamic characteristics of genomic loci and chromosomes, as observed in live-cell imaging experiments, and allow Hi-C data to be interpreted as dynamic information on the 3D genome organization (Shinkai *et al.*, 2020a). However, although the reconstructed contact matrix from an input contact matrix shows excellent agreement with the Pearson correlation coefficient (PCC) of more than 95% (Shinkai *et al.*, 2020c), the optimization procedure, which is a core part of the PHi-C algorithm, is a computational bottleneck; the iterative algorithm to reduce the cost function at every optimization step requiring several days to obtain an optimal solution. At each step, a randomly selected matrix element is slightly changed; moreover, all matrix elements are needed to calculate the cost function. This redundant computational algorithm is inefficient. Furthermore, by defining the cost function according to the logarithmic form during optimization and interpolation for the null value of an input contact matrix, PHi-C is not appropriate for every Hi-C matrix data.

To overcome these problems, we first found the mathematical transformation between an input contact matrix and a set of parameters of our polymer model, and the forward and inverse transformations were in the invertible correspondence (Supplementary Note S1). Then, we elucidated the mathematical concept of the optimization and updated the optimization algorithm (Supplementary Note S2). Benchmarks of the optimization calculations indicated that the scores were improved in terms of both the speed and closeness between the input and optimal contact matrices (Supplementary Note S3). In addition, we incorporated a rheology analysis (Shinkai *et al.*, 2020a) as a new function to convert Hi-C data into the spectrum of the dynamic rheological properties along the genomic coordinate of a single chromosome (Supplementary Note S4). Here, we present PHi-C2 as a Python package, redesigned from the ground up with additional new features. Additionally, we included a command-line interface (CLI) for convenient application.

## 2 Implementation and benchmarks

**Fig. 1.**
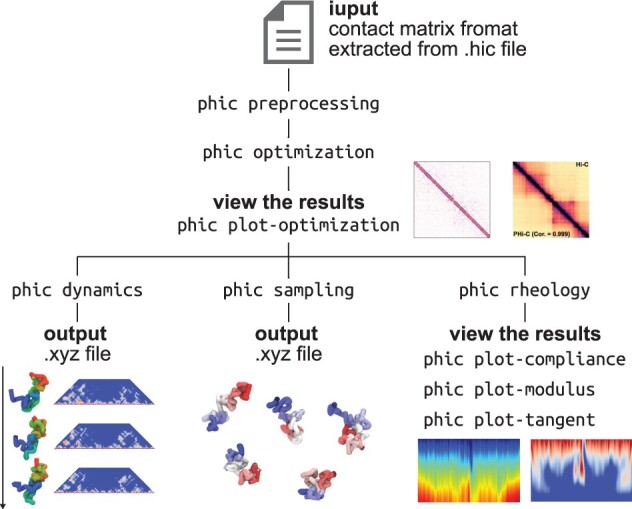
Overview of the PHi-C2 pipeline and phic CLI commands. PHi-C2 analyzes input Hi-C data as the contact matrix format extracted from .hic file by Juicer and Straw (Durand *et al.*, 2016). The updated optimization algorithm outputs an optimal parameter set for the polymer model and depicts the optimal contact matrix. Using the optimal parameters, users can calculate the polymer model’s dynamics, structure sampling and rheology spectra, which are consistent with the input Hi-C matrix

PHi-C2 is implemented using the phic Python package, which includes a suite of CLI subcommands under a top-level phic command namespace (Fig. 1). The input Hi-C file is the contact matrix format extracted from the .hic file by Juicer and Straw (Durand *et al.*, 2016). First, the phic preprocessing command converts the input into the normalized contact matrix data so that the diagonal elements are units based on the PHi-C polymer modeling theory. Next, the phic optimization command outputs an optimal matrix as the PHi-C polymer model parameter set. To visualize the results of a reconstructed contact matrix and a contact probability decay curve, we prepared the phic plot-optimization command. After the optimization procedure, users can calculate the polymer model’s dynamics and structure sampling using the phic dynamics and phic sampling commands, respectively. The outputs are .xyz and .psf format files, and visualization requires VMD (Humphrey *et al.*, 1996). Furthermore, to reveal the hierarchical and dynamic 3D genome state embedded in the input 2D Hi-C pattern, users can apply the phic rheology command and the three consecutive commands (phic plot-compliance, phic plot-modulus and phic plot-tangent) to visualize the rheological analysis results. In addition, without introducing a Python environment in the user’s local platform, PHi-C2 can run on Google Colab, where users can easily change parameters and check the results of the plots along the workflow in the notebook.

As the optimization procedure is a core computational part of PHi-C2, we benchmarked the performance for a 400 × 400-sized input Hi-C matrix (chr1: 50–60 Mb, 25-kb bins) of mouse embryonic stem cells (Bonev *et al.*, 2017) (Supplementary Note S3). First, the updated PHi-C2 algorithm improved the speed and accuracy compared to that obtained using the previous PHi-C version (Supplementary Fig. S3). By varying the optimization parameters in terms of the initial values, learning rate, and stop condition, we obtained optimal solutions with the PCC, r≥0.997, and distance-corrected PCC (Bianco *et al.*, 2018), r′≥0.957, between the input and optimized contact matrices (Supplementary Table S2). All tests were finished in 30 min for an Intel^®^ Xeon^®^ Gold 6154 processor (24.75 M Cache, 3.00 GHz) with Intel^®^ distribution for the Python environment and in 90 min for the Google Colab environment.

Note that PHi-C2’s input contact matrix should be denser because all the null contact elements in a binning resolution are regarded as zeros. However, the null element is not necessarily equivalent to the zero value of the contact probability. Although the null-value contribution has been eliminated when calculating the cost function (Liu *et al.*, 2021), PHi-C2 has not implemented it. The iterative optimization process depends on the input matrix size (Shinkai *et al.*, 2020b), while 100 × 100–500 × 500 is a good and practical input matrix size according to the genomic region of interest (Supplementary Table S3). Users need to appropriately adjust the binning resolution and genomic region for the input.

## 3 Conclusion

We developed a Python package, PHi-C2, to analyze Hi-C matrix data, including CLI subcommands for convenient manipulation. As we reconsidered the mathematical framework and eliminated the computational bottleneck of the previous version, the speed and accuracy improved. Therefore, without a massive computational cost, users can calculate the polymer dynamics, structural conformations, and rheological features consistent with the input Hi-C data. The easy installation from the Python package index and calculations on Google Colab would help users reveal the physical features embedded in Hi-C data.

## Supplementary Material

btac613_Supplementary_Data

## Data Availability

The data underlying this article are available in the article and in its online supplementary material.
