# Supplementary material for: PHi-C2: interpreting Hi-C data as the dynamic 3D genome state
Source: Bioinformatics. 2022 Sep 10;38(21):4984–6. doi: 10.1093/bioinformatics/btac613 (PMC9620818; doi:10.1093/bioinformatics/btac613)
Supplement: btac613_Supplementary_Data [file btac613_supplementary_data.pdf]

SUPPLEMENTARY INFORMATION
PHi-C2: interpreting Hi-C data as the dynamic 3D genome state

Soya Shinkai[1], **Hiroya Itoga**[1], **Koji Kyoda**[1], **and Shuichi Onami**[1,2]

[1]Laboratory for Developmental Dynamics, RIKEN Center for Biosystems Dynamics Research, Kobe, 650-0047, Japan.
[2]Life Science Data Sharing Unit, Infrastructure Research and Development Division, RIKEN Information R&D and Strategy Headquarters, Kobe, 650-0047, Japan.

ABSTRACT

Supplementary information includes 4 Supplementary Notes, 5 Supplementary Figures, and 3 Supplementary Tables.

Supplementary Note S1: Inverse transformation to reconstruct an input Hi-C matrix

PHi-C is a simulation tool to decipher Hi-C data into polymer dynamics based on the mathematical formalism of the polymer network model[1]. The normalized interaction matrix $\bar{\mathsf{K}} = \left(\bar{K}_{ij}\right)$ of the model has a one-to-one correspondence to a normalized contact matrix $\bar{\mathsf{C}} = \left(\bar{C}_{ij}\right)$ through the following matrix transformations, where the matrix size is $N \times N$: (i) $\bar{\mathsf{K}}$ is converted into the normalized Laplacian matrix $\bar{\mathsf{L}} = \left(L_{ij}\right)$ by $\bar{\mathsf{L}} = \bar{\mathsf{D}} - \bar{\mathsf{K}}$, with the normalized degree matrix $\bar{\mathsf{D}} = \mathrm{diag}(\bar{D}_0, \bar{D}_1, ..., \bar{D}_{N-1})$, where $\bar{D}_i = \sum_{j=0}^{N-1} \bar{K}_{ij}$. (ii) As the Laplacian matrix $\bar{\mathsf{L}}$ is real symmetric, $\bar{\mathsf{L}}$ is diagonalizable. In addition, the N eigenvalues satisfy $0 = \bar{\lambda}_0 < \bar{\lambda}_1 < \cdots < \bar{\lambda}_{N-1}$, as long as $\bar{\mathsf{L}}$ is positive semidefinite and there is an orthogonal matrix Q such that $\mathsf{Q}^\mathsf{T}\bar{\mathsf{L}}\mathsf{Q} = \mathrm{diag}(0, \bar{\lambda}_1, \cdots, \bar{\lambda}_{N-1})$. Then, $\bar{\mathsf{L}}$ is transformed into the normalized covariance matrix $\bar{\mathsf{M}} = \left(\bar{M}_{ij}\right)$ by $\bar{\mathsf{M}} = \mathsf{Q}\,\mathrm{diag}\left(0, \bar{\lambda}_1^{-1}, \cdots, \bar{\lambda}_{N-1}^{-1}\right)\mathsf{Q}^\mathsf{T}$. (iii) $\bar{\mathsf{M}}$ is converted into the normalized variance matrix $\bar{\Sigma}^2 = \left(\bar{\Sigma}^2_{ij}\right) = \left(\frac{\bar{M}_{ii} + \bar{M}_{jj} - 2\bar{M}_{ij}}{3}\right)$. (iv) $\bar{\Sigma}^2$ is transformed into the contact matrix $\mathsf{C} = \left(1 + \bar{\Sigma}^2\right)^{-3/2}$. Here, we briefly express this procedure as $\mathsf{C} = \varphi(\bar{\mathsf{K}})$. If we can find the inverse function φ^{-1}, we can solve the inverse problem $\bar{\mathsf{K}} = \varphi^{-1}(\mathsf{C})$ for any input contact matrix.

The first step of the inverse function is $\bar{\Sigma}^2 = \left(\mathsf{C}^{-2/3} - 1\right)$. As we previously noted[1], there is an inverse transformation from $\bar{\Sigma}^2$ to $\bar{\mathsf{M}}$ because the matrix $\bar{\mathsf{M}}$ satisfies the condition $\bar{\mathsf{M}}\mathbf{1} = \mathbf{0}$, where $\mathbf{1}$ is a vector $(1, 1, \cdots, 1)^\mathsf{T}$. Here, we explicitly provide the inverse transformation. Let $\boldsymbol{m}$ be a vector that consists of the diagonal elements of the matrix $\bar{\mathsf{M}}$, $\left(\bar{M}_{00}, \bar{M}_{11}, \cdots, \bar{M}_{N-1,N-1}\right)^\mathsf{T}$, and we define a square matrix B as $(\boldsymbol{m}, \boldsymbol{m}, \cdots, \boldsymbol{m})$. Then, the relationship between $\bar{\Sigma}^2$ and $\bar{\mathsf{M}}$ can be written as $3\bar{\Sigma}^2 = \mathsf{B} + \mathsf{B}^\mathsf{T} - 2\bar{\mathsf{M}}$. Multiplying $\mathbf{1}$ from the right, we obtain $3\bar{\Sigma}^2\mathbf{1} = \mathsf{A}\boldsymbol{m}$, where A is a square matrix defined by $N\mathsf{I} + \mathbf{1}\mathbf{1}^\mathsf{T}$. Since the matrix A is non-singular, there exists the inverse matrix given by $\mathsf{A}^{-1} = \frac{2N\mathsf{I} - \mathbf{1}\mathbf{1}^\mathsf{T}}{2N^2}$. Therefore, we can determine the diagonal elements of the matrix $\bar{\mathsf{M}}$ by $\boldsymbol{m} = 3\mathsf{A}^{-1}\bar{\Sigma}^2\mathbf{1}$ and calculate all elements by $\bar{M}_{ij} = \frac{\bar{M}_{ii} + \bar{M}_{jj} - 3\bar{\Sigma}^2_{ij}}{2}$.

Next, we considered a transformation from $\bar{\mathsf{M}}$ to $\bar{\mathsf{L}}$. Theoretically, the matrix $\bar{\mathsf{M}}$ should be positive semidefinite according to the definition. However, $\bar{\mathsf{M}}$ calculated through the above transformation did not necessarily satisfy the positive semidefiniteness requirement, which is mainly due to the experimental noise of Hi-C data. Therefore, the Laplacian matrix $\bar{\mathsf{L}}$ could not be found in a straightforward manner by solving the eigenvalue problem. Instead, a different strategy was used to construct a pseudo-Laplacian matrix $\tilde{\mathsf{L}}$ using the Moore-Penrose (MP) inverse matrix $\bar{\mathsf{M}}^+$ of the matrix $\bar{\mathsf{M}}$[2]. Matrices $\bar{\mathsf{M}}$ and $\bar{\mathsf{L}}$ theoretically satisfy the relationship $\bar{\mathsf{M}}\bar{\mathsf{L}} = \mathsf{I} - \frac{\mathbf{1}\mathbf{1}^\mathsf{T}}{N}$; therefore, the MP inverse matrix minimizes the Frobenius norm $\left\|\bar{\mathsf{M}}\bar{\mathsf{L}} - \mathsf{I} + \frac{\mathbf{1}\mathbf{1}^\mathsf{T}}{N}\right\|_\mathrm{F}$ and provides the solution by $\tilde{\mathsf{L}} = \bar{\mathsf{M}}^+\left(\mathsf{I} - \frac{\mathbf{1}\mathbf{1}^\mathsf{T}}{N}\right)$. The construction of the MP inverse matrix is based on the singular value decomposition of the matrix $\bar{\mathsf{M}}$, and we can easily implement it on numerical scripts. Finally, we can obtain a pseudo-interaction matrix $\tilde{\mathsf{K}}$ by $\tilde{\mathsf{K}} = -\tilde{\mathsf{L}}$ and zero-diagonals ($\tilde{K}_{ii} = 0$).

Based on the above algorithm, we reconstructed a contact matrix $\tilde{\mathsf{C}} = \varphi(\tilde{\mathsf{K}})$ from an input matrix C (Supplementary Figure S1A). Note that although the pseudo-Laplacian matrix $\tilde{\mathsf{L}}$ is no longer positive semidefinite (Supplementary Figure S1B), the numerical calculation of the eigenvalues and eigenvectors proceeds normally. We estimated the closeness of all elements between C and $\tilde{\mathsf{C}}$. Surprisingly, the `allclose` function in NumPy returned `True`, which means that the two matrices are element-wise equal within the default numerical tolerance. Taken together, we were able to find the inverse transformation φ^{-1}.

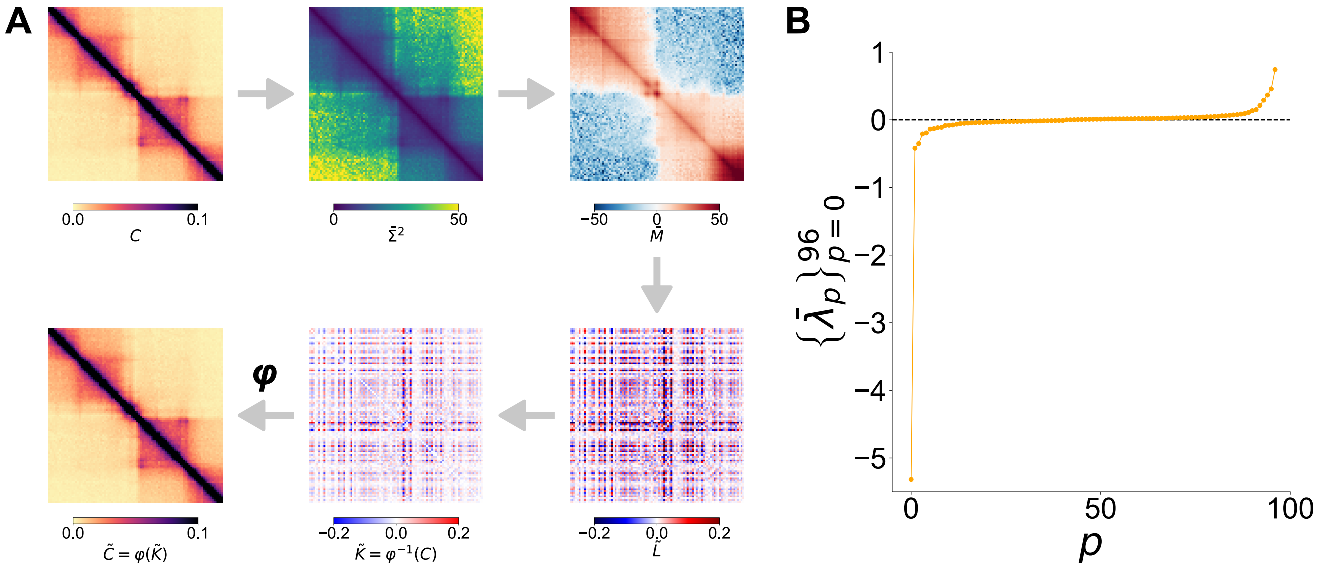

Supplementary Figure S1. Matrix transformations in PHi-C polymer modeling. **(A)** Inverse transformation φ^{-1} converts an input normalized contact matrix C [chr8: 42,100–44,525 kb (25-kb bins) for mouse embryonic stem cells (mESCs)[3]] into the pseudo-interaction matrix $\tilde{\mathsf{K}}$ via matrices $\bar{\Sigma}^2$, $\bar{\mathsf{M}}$ and $\tilde{\mathsf{L}}$. The reconstructed contact matrix $\tilde{\mathsf{C}} = \varphi(\tilde{\mathsf{K}})$ and the input contact matrix C are equal within the default numerical tolerance. **(B)** Eigenvalues of the pseudo-Laplacian matrix $\tilde{\mathsf{L}}$. Since there are negative eigenvalues, $\tilde{\mathsf{L}}$ is no longer positive semidefinite.

Supplementary Note S2: Updated optimization operation

Mathematical concept of the PHi-C optimization

The optimization procedure is a central part of PHi-C. We define the optimization process as an iterative finding of the normalized interaction matrix $\bar{\mathsf{K}}$ that decreases a cost function $f(\mathsf{C}, \varphi(\bar{\mathsf{K}}))$ for an input contact matrix C. To clarify the mathematical concept, we introduce the following sets (Supplementary Figure S2):

- $\mathbb{K}^N = \left\{ \bar{\mathsf{K}} | \text{ a symmetric } N \times N \text{ matrix with zero diagonals } \bar{K}_{ii} = 0 \right\}$,
- $U_\epsilon = \left\{ \bar{\mathsf{K}} \in \mathbb{K}^N | f(\mathsf{C}, \varphi(\bar{\mathsf{K}})) < \epsilon \right\}$,
- $A = \left\{ \bar{\mathsf{K}} \in \mathbb{K}^N | \text{ the induced Laplacian matrix } \bar{\mathsf{L}} \text{ is positive-semidefinite and } \bar{K}_{i,i+1} > 0 \right\}$.

For a small ϵ, a matrix $\bar{\mathsf{K}} \in U_\epsilon$ is an optimal candidate. In Supplementary Note 1, we found the minimal solution $\varphi^{-1}(\mathsf{C}) = \tilde{\mathsf{K}} \in U_{\epsilon=0}$ by the inverse transformation. However, since the induced Laplacian matrix does not necessarily satisfy the positive semidefiniteness, the polymer system becomes unstable and the normalized interaction matrix $\tilde{\mathsf{K}}$ is unrealistic. Thus, the set A ensures the physically acceptable matrix $\bar{\mathsf{K}}$ with connections along the polymer backbone $\bar{K}_{i,i+1} > 0$ as a stable polymer system.

Let $\bar{\mathsf{K}}_n$ be the normalized interaction matrix at the n-th iteration step in the optimization procedure. An initial matrix $\bar{\mathsf{K}}_0$ should be in the set A, and an optimization path in $\mathbb{K}^N$ would go pass into set U_ϵ for a small ϵ (Supplementary Figure S2). Then, paths that output an optimal $\bar{\mathsf{K}} \in U_\epsilon \cap A$ are acceptable, while paths that pass into $U_\epsilon \cap A^c$ should be forbidden.

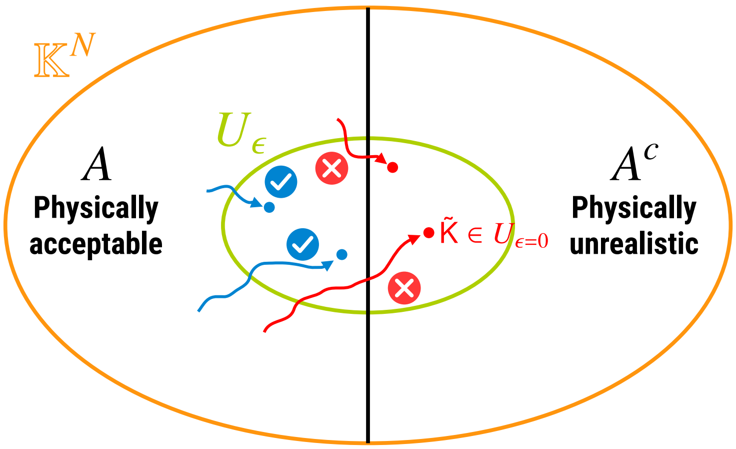

Supplementary Figure S2. Mathematical concept of the PHi-C optimization. The PHi-C optimization iteratively decreases the cost function in $\mathbb{K}^N$. The set U_ϵ has optimal candidates of the matrix $\bar{\mathsf{K}}$. As the inverse transformation induces the pseudo-interaction matrix $\tilde{\mathsf{K}}$, the matrix $\tilde{\mathsf{K}}$ is just an element of the set $U_{\epsilon=0}$. Optimization paths can pass into the set $U_\epsilon \cap A^c$, where the polymer systems are physically unrealistic. Optimization paths should move in the physically acceptable set A.

Updated algorithm

We mainly updated the optimization algorithm regarding the definition of the cost function and the iterative operation at each step (Supplementary Table S1). Accordingly, both factors provided faster and more accurate optimization results than the previous version.

First, we re-defined the cost function as

$$f(\mathsf{C}, \varphi(\bar{\mathsf{K}})) = \sqrt{\frac{1}{N^2} \sum_{i=0}^{N-1} \sum_{j=0}^{N-1} \left[C_{ij} - (\varphi(\bar{\mathsf{K}}))_{ij} \right]^2}.$$

	PHi-C1	PHi-C2
Cost function: $f(\mathsf{C},\varphi(\bar{\mathsf{K}}))$	$\sqrt{\frac{1}{N^2}\sum_{i=0}^{N-1}\sum_{j=0}^{N-1}\left[\log_{10}C_{ij}-\log_{10}(\varphi(\bar{\mathsf{K}}))_{ij}\right]^2}$	$\sqrt{\frac{1}{N^2}\sum_{i=0}^{N-1}\sum_{j=0}^{N-1}\left[C_{ij}-(\varphi(\bar{\mathsf{K}}))_{ij}\right]^2}$
Optimization operation at every iteration step	For a randomly selected pair (i, j),<br>$\xi = \eta \times \text{random.uniform}(0, 1)$<br>**if** $\log_{10}(\varphi(\bar{\mathsf{K}}_{n-1}))_{ij} > \log_{10} C_{ij}$ **then**<br>$(\bar{\mathsf{K}}_n)_{ij} = (\bar{\mathsf{K}}_{n-1})_{ij} - \xi$<br>$(\bar{\mathsf{K}}_n)_{ji} = (\bar{\mathsf{K}}_{n-1})_{ji} - \xi$<br>**else**<br>$(\bar{\mathsf{K}}_n)_{ij} = (\bar{\mathsf{K}}_{n-1})_{ij} + \xi$<br>$(\bar{\mathsf{K}}_n)_{ji} = (\bar{\mathsf{K}}_{n-1})_{ji} + \xi$	For all the elements,<br>$\bar{\mathsf{K}}_n = \bar{\mathsf{K}}_{n-1} - \eta\left(\varphi(\bar{\mathsf{K}}_{n-1}) - \mathsf{C}\right)$

Supplementary Table S1. Mainly updated algorithms of PHi-C2 compared to PHi-C1. η is the learning rate parameter in the optimization operation.

This value represents the standard deviation between the contact probabilities C and $\varphi(\bar{\mathsf{K}})$ per element. Moreover, we calculated the standard deviation between the logarithmic contact probabilities in the previous version (Supplementary Table S1). Since the logarithmic function is not defined for a zero value, an interpolate operation is needed for the zero-valued elements to ensure that the shape of the contact probability curve remains unaltered[1]. Therefore, the newly defined cost function does not require the interpolation process.

Every iterative optimization step updates all the elements of the matrix $\bar{\mathsf{K}}_n$ and obeys the following rule:

$$\bar{\mathsf{K}}_n = \bar{\mathsf{K}}_{n-1} - \eta\left(\varphi(\bar{\mathsf{K}}_{n-1}) - \mathsf{C}\right),$$

where η is the learning rate parameter. In the previous version of PHi-C, the random choice of a matrix element (i, j) and its slight change of the element value at every iteration step were a bottleneck (Supplementary Table S1). The new iterative rule overcomes the bottleneck by updating the values of all the elements at one time.

Finally, we set a stop condition due to a monotonical decrease of the cost function,

$$0 < f(\mathsf{C},\varphi(\bar{\mathsf{K}}_{n-1})) - f(\mathsf{C},\varphi(\bar{\mathsf{K}}_n)) < \delta \quad \Longrightarrow \quad \text{output } \bar{\mathsf{K}}_n \text{ as an optimal solution } \bar{\mathsf{K}}_{\text{opt}},$$

where $\delta = \eta\alpha$ and α is a parameter for the stop condition.

Overall, the above three points are significant updates of the PHi-C2 algorithm. Thus far, we have not been able to mathematically ensure that every optimization path decreases the cost function monotonically in the set A (Supplementary Figure S2). The update coefficient $\left(\varphi(\bar{\mathsf{K}}_{n-1}) - \mathsf{C}\right)$ does not exactly correspond to the gradient $\left.\frac{\partial f(\mathsf{C},\varphi(\bar{\mathsf{K}}))}{\partial \bar{K}_{ij}}\right|_{\bar{\mathsf{K}}=\bar{\mathsf{K}}_{n-1}}$, although the gradient-based algorithm has already been formulated[4] and shows a good performance[5]. However, we can operationally obtain an optimal solution in the set A by tuning the initial point $\bar{\mathsf{K}}_0 \in A$, learning rate η, and stop condition parameter α.

Supplementary Note S3: Benchmarks

After the optimization process, we can estimate the closeness of the optimal contact matrix $\mathsf{C}_{\rm opt} = \varphi(\bar{\mathsf{K}}_{\rm opt})$ to the input contact matrix C using the Pearson correlation coefficient (PCC), r, and distance-corrected PCC[6], r', for the non-diagonal elements of both matrices. Here, we show benchmarks for Hi-C data of mESCs[3] [chr1: 50–60 Mb (25-kb bins)].

For the 400×400-sized input matrix of mESCs and a default set of optimization parameters ($(\bar{\mathsf{K}}_0)_{i,i+1} = 0.5$, $\eta = 10^{-4}$, $\alpha = 10^{-4}$), PHi-C2 output an optimal solution with $r = 0.998$ and $r' = 0.970$ in 26,307 iteration steps (Supplementary Figure S3A and Supplementary Table S2). The differences between the PHi-C1 and PHi-C2 optimization algorithms are summarized in Supplementary Table S1, and they indicate that the calculation of the cost function $f(\mathsf{C}, \phi(\bar{\mathsf{K}}_n))$ for all matrix elements in PHi-C1 seems relatively inefficient for a slight change of a randomly selected matrix element at each iterative step. In contrast, all matrix elements are updated together using the feature of NumPy for matrix calculations in PHi-C2. Therefore, the optimization process should be more efficient. As expected, for the same iteration steps and $\eta = 4 \times 10^{-3}$, an optimal solution of the previous PHi-C (PHi-C1) had a value of $r = 0.966$ (Supplementary Figure S3B), although the meaning of the cost function and the learning rate differs between PHi-C1 and PHi-C2. We can confirm that the updated algorithm improves the speed and accuracy of the optimization procedure compared to that of PHi-C1.

Next, we show benchmarks for different optimization parameters: initial values of the polymer backbone $(\bar{\mathsf{K}}_0)_{i,i+1}$, learning rate η, and stop condition parameter α (Supplementary Table S2). We carried out all calculations using an Intel® Xeon® Gold 6154 processor (24.75 M Cache, 3.00 GHz) with Intel® distribution for the Python environment and Google Colab environment. The procedure stopped within less than 100,000 iteration steps and 30 min and 90 min for these parameters in our workstation and the Google Colab environment, respectively. Interestingly, although the optimal cost function values differed, the correlation values reached 0.998. As our stop condition relates to the cost function curve saturation, the optimal solutions would be close to local minimums in the set $U_{\varepsilon=0.00223}$. Also, we could confirm that the optimal solutions are located in the set A due to the positive semidefiniteness (Supplementary Figure S3C).

The speed of the PHi-C2 optimization depends on the matrix calculations at every iterative step. To clarify the speed and accuracy features, we benchmarked the PHi-C2 optimization by changing the different bin sizes from 25 kb to 50, 100, and 250 kb for the same genomic region [chr1: 50–60 Mb] (Supplementary Table S3). The optimized accuracy and number of iterative steps differed due to the different input Hi-C matrices. At the same time, the calculation time dramatically decreased depending on the reduction of the matrix size. A rough estimation of the ratio (9m54s)/(2m41s) $= 3.69\cdots \simeq 4$ for 25- and 50-kb bin sizes implies that the optimization time is in $O(N^2)$, where N is the number of the diagonal bins or beads of our polymer model.

As we stated, the update algorithm does not always decrease the cost function monotonically. We observed such counter-examples for Hi-C data of the mouse erythroid cells[7] [chr1: 3–195.5 Mb (500-kb bins)] in the prometaphase. Here, we altered only the initial values of the polymer backbone $(\bar{\mathsf{K}}_0)_{i,i+1} = 0.1 \sim 1.0$ (fixed $\eta = 10^{-4}$ and $\alpha = 10^{-4}$). Some curves showed an increase in the cost function within the initial 100 steps (Supplementary Figure S4A). Nevertheless, every curve eventually converged and output an optimal solution. Moreover, for Hi-C data in the late G1 phase, we confirmed a monotonical decrease of the cost function (Supplementary Figure S4B).

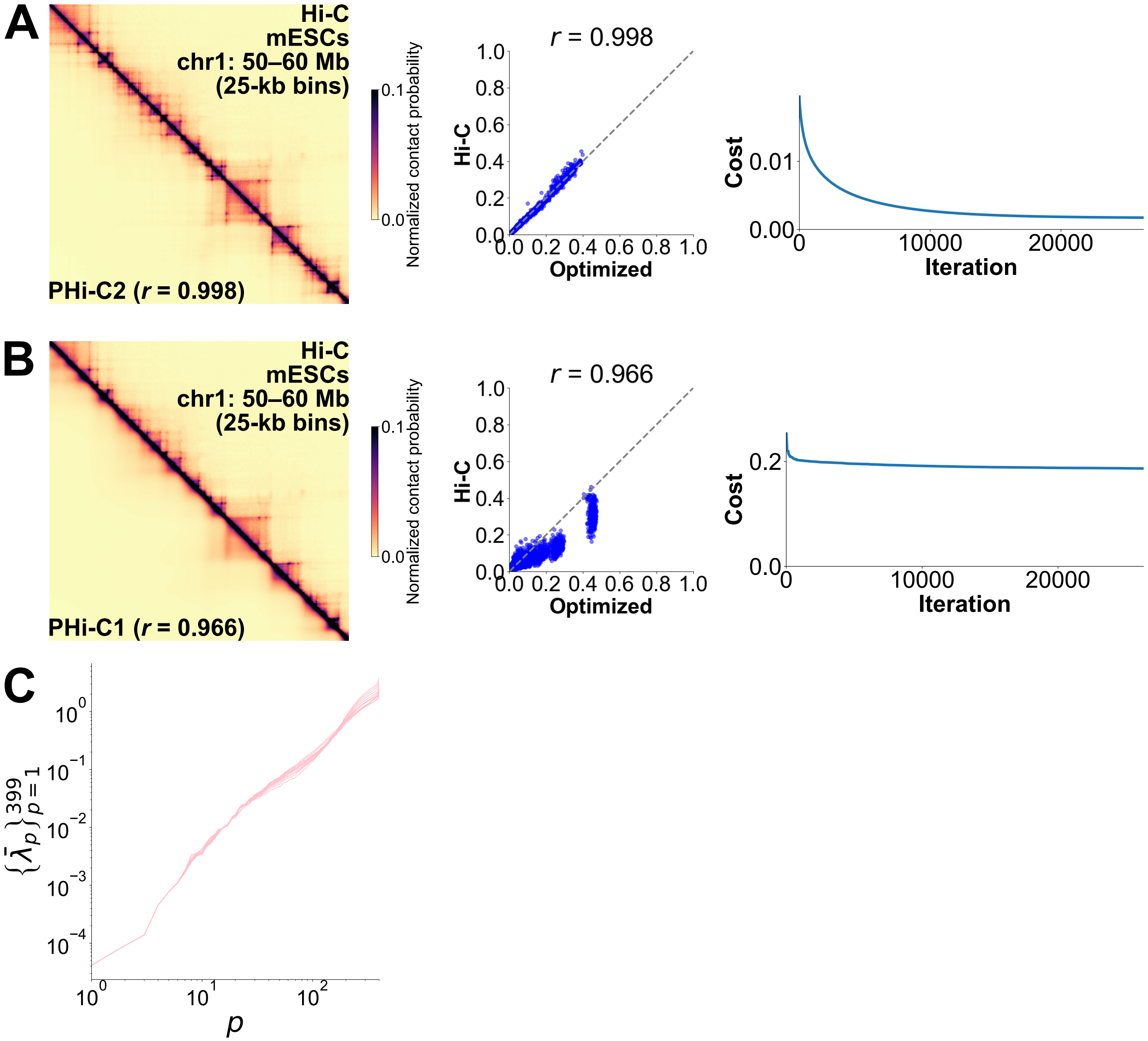

Supplementary Figure S3. Comparison between (A) PHi-C2 and (B) PHi-C1. We used Hi-C data of mESCs [chr1: 50–60 Mb (25-kb bins)] as input. (Left) Heatmap of the contact matrix consists of the input (upper-right) and the optimal (lower-left) matrices. (Middle) Scatter plot with the PCC r visualizes the closeness of the input and optimized contact matrices. (Right) Curve of the cost function in the optimization steps. **(C)** Eigenvalues of the induced Laplacian matrix from the optimal solution are positive except for $\bar{\lambda}_0 = 0$. We plotted eigenvalue spectra for the optimal solutions in Supplementary Table S2.

$\left(\bar{\mathsf{K}}_0\right)_{i,i+1}$	η	α	# iterations	time	time (Colab)	$f(\mathsf{C},\varphi(\bar{\mathsf{K}}_{\text{opt}}))$ [$\times 10^{-3}$]	r	r'
0.1	10^{-4}	10^{-4}	68,164	24 m 27 s	88 m 13 s	2.10086	0.998	0.982
0.2	10^{-4}	10^{-4}	57,477	20 m 52 s	72 m 51 s	2.09129	0.998	0.980
0.3	10^{-4}	10^{-4}	36,388	13 m 21 s	47 m 15 s	2.11411	0.998	0.976
0.4	10^{-4}	10^{-4}	17,931	7 m 00 s	20 m 56 s	1.94762	0.997	0.966
0.5	10^{-4}	10^{-4}	26,307	9 m 54 s	32 m 7 s	1.73502	0.998	0.970
0.6	10^{-4}	10^{-4}	35,097	13 m 48 s	43 m 46 s	1.62454	0.998	0.972
0.7	10^{-4}	10^{-4}	43,714	16 m 51 s	54 m 46 s	1.56127	0.998	0.973
0.8	10^{-4}	10^{-4}	52,089	19 m 54 s	64 m 39 s	1.52338	0.998	0.974
0.9	10^{-4}	10^{-4}	60,228	20 m 48 s	77 m 54 s	1.50057	0.998	0.975
1.0	10^{-4}	10^{-4}	68,139	24 m 34 s	75 m 0 s	1.48751	0.998	0.976
0.5	2×10^{-4}	10^{-4}	13,154	4 m 24 s	16 m 43 s	1.73501	0.998	0.970
0.5	10^{-4}	10^{-4}	26,307	9 m 54 s	32 m 7 s	1.73502	0.998	0.970
0.5	5×10^{-5}	10^{-4}	52,613	17 m 35 s	63 m 19 s	1.73502	0.998	0.970
0.5	10^{-4}	10^{-3}	13,369	4 m 31 s	16 m 16 s	2.22941	0.997	0.957
0.5	10^{-4}	10^{-4}	26,307	9 m 54 s	32 m 7 s	1.73502	0.998	0.970
0.5	10^{-4}	10^{-5}	42,895	14 m 15 s	48 m 33 s	1.67631	0.998	0.976

Supplementary Table S2. Benchmarks of PHi-C2 for different optimization parameters. We used Hi-C data of mESCs [chr1: 50–60 Mb (25-kb bins)].

Bin size	Matrix size	# iterations	time	$f(\mathsf{C},\varphi(\bar{\mathsf{K}}_{\text{opt}}))$ [$\times 10^{-3}$]	r	r'
25 kb	400×400	26307	9 m 54 s	1.73502	0.998	0.970
50 kb	200×200	41978	2m 41 s	1.57381	0.999	0.988
100 kb	100×100	63261	60 s	1.93041	0.999	0.991
250 kb	40×40	79840	18 s	3.80607	0.998	0.982

Supplementary Table S3. Benchmarks of PHi-C2 for different matrix sizes. We used Hi-C data of mESCs [chr1: 50–60 Mb] and set default initial optimization parameters ($(\bar{\mathsf{K}}_0)_{i,i+1} = 0.5$, $\eta = 10^{-4}$, $\alpha = 10^{-4}$).

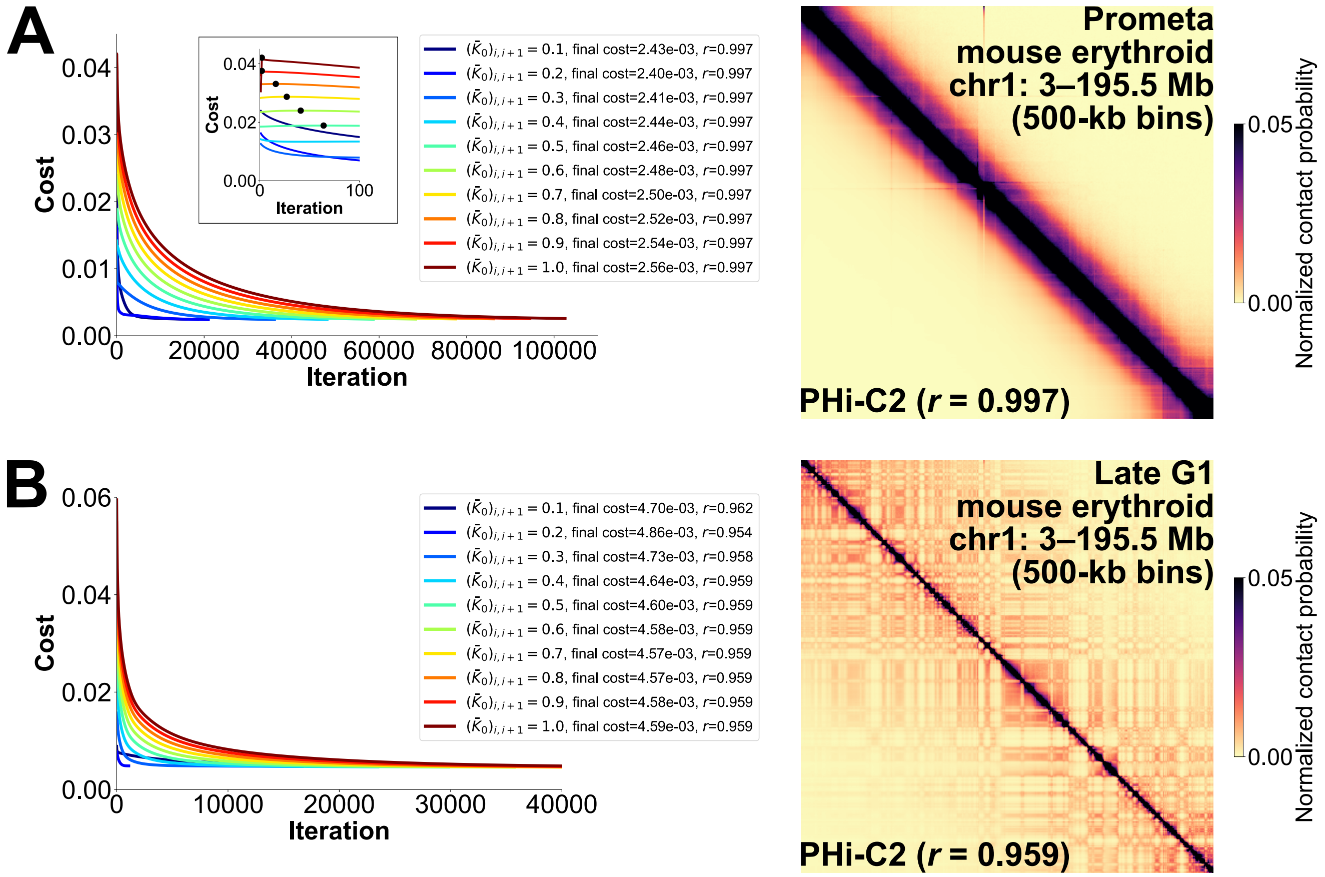

Supplementary Figure S4. PHi-C2 results for the (A) prometaphase and (B) late G1 phase. We used Hi-C data of mouse erythroid cells [chr1: 3–195.5 Mb (500-kb bins)] as the input. (Left) Curves of the cost function. (Right) Heatmap of the contact matrix that consists of the input (upper-right) and optimal (lower-left) matrices. **(A)** (Inset) Magnification of the initial 100 iteration steps. The black points represent the local maximum point for $(\bar{K}_0)_{i,i+1} = 0.5 \sim 1.0$. The heatmap consists of the input contact matrix (upper-right) and the optimal matrix (lower-left) for $(\bar{K}_0)_{i,i+1} = 0.2$. **(B)** The heatmap consists of the input contact matrix (upper-right) and the optimal matrix (lower-left) for $(\bar{K}_0)_{i,i+1} = 0.7$.

Supplementary Note S4: Rheology analysis as an additional function

We added rheology analysis commands as a new function in PHi-C2. This new feature provides a method of interpreting the spatial hierarchy of the 3D genome organization as dynamic 3D genome information[8]. For example, we could conclude that TAD boundaries behave as more rigid nodes than intra-TAD regions. Using the algorithm, we can convert the optimal solution $\bar{\mathsf{K}}_{\mathrm{opt}}$ into the rheology spectra of the normalized complex compliance $\bar{J}^*$, the normalized complex modulus $\bar{G}^*$, and the loss tangent $\tan\delta$.

In soft matter physics, the complex compliance $J^*(\omega)$ for periodic mechanical stress with the angular frequency ω is the ratio of strain to stress[9]. In addition, the complex compliance possesses real and imaginary components: $J^*(\omega) = |J^*(\omega)|e^{-i\delta(\omega)} = J'(\omega) - iJ''(\omega)$. The real component is the storage compliance related to the elastic response, while the imaginary component is the loss compliance as the viscous response. Therefore, the ratio $J''(\omega)/J'(\omega)$ corresponds to $\tan\delta(\omega)$ and is a measure of liquid-like ($\tan\delta(\omega) > 1$) or solid-like ($\tan\delta(\omega) < 1$) behavior. Furthermore, the complex modulus $G^*(\omega) = |G^*(\omega)|e^{i\delta(\omega)} = G'(\omega) + iG''(\omega)$ relates to the complex compliance by $G^*(\omega) = 1/J^*(\omega)$.

Here, we outline the calculation of the complex compliance[8]. Note that in PHi-C1 and PHi-C2, the spatial and temporal scales of all physical variables are normalized by combining the contact distance σ, friction coefficient of the beads γ, Boltzmann constant k_{B}, and temperature T. First, we diagonalize the normalized Laplacian matrix $\bar{\mathsf{L}}$ induced from the normalized optimal solution $\bar{\mathsf{K}}_{\mathrm{opt}}$ to obtain the orthogonal matrix Q and the normalized eigenvalues $0 < \bar{\lambda}_0 < \bar{\lambda}_1 < \cdots < \bar{\lambda}_{N-1}$ that satisfy $\mathsf{Q}^{\mathsf{T}}\bar{\mathsf{L}}\mathsf{Q} = \mathrm{diag}(\bar{\lambda}_0, \bar{\lambda}_1, \cdots, \bar{\lambda}_{N-1})$. Then, we derive the normalized complex compliance by

$$\bar{J}^*(\bar{\omega}; n) = \sum_{p=1}^{N-1} \frac{Q_{np}^2}{3\bar{\lambda}_p + i\bar{\omega}}.$$

Therefore, the normalized storage and loss compliances can be given as

$$\bar{J}'(\bar{\omega}; n) = \sum_{p=1}^{N-1} \frac{3Q_{np}^2\bar{\lambda}_p}{9\bar{\lambda}_p^2 + \bar{\omega}^2} \quad \text{and} \quad \bar{J}''(\bar{\omega}; n) = \sum_{p=1}^{N-1} \frac{Q_{np}^2\bar{\omega}}{9\bar{\lambda}_p^2 + \bar{\omega}^2},$$

respectively. Since n represents the index of the beads or bins ($n = 0, 1, \cdots, N-1$) along the genomic coordinate, we can plot a two-dimensional heatmap as the rheology spectrum of $|\bar{J}^*(\bar{\omega}; n)|$ and arrange it under the input Hi-C pattern map. The normalized complex modulus $\bar{G}^*(\bar{\omega}; n)$ and the loss tangent $\tan\delta(\bar{\omega}; n)$ can be calculated from the following definitions: $\bar{G}^*(\bar{\omega}) = 1/\bar{J}^*(\omega)$ and $\tan\delta(\bar{\omega}) = \bar{J}''(\bar{\omega})/\bar{J}'(\bar{\omega})$.

Here, we show examples for the Hi-C data of mESCs[3] [chr17: 3–95 Mb (250-kb bins), chr17: 41–59.4 Mb (50-kb bins), and chr17: 49–52.68 Mb (10-kb bins)] (Supplementary Figure S5). We fixed the matrix size to 368×368.

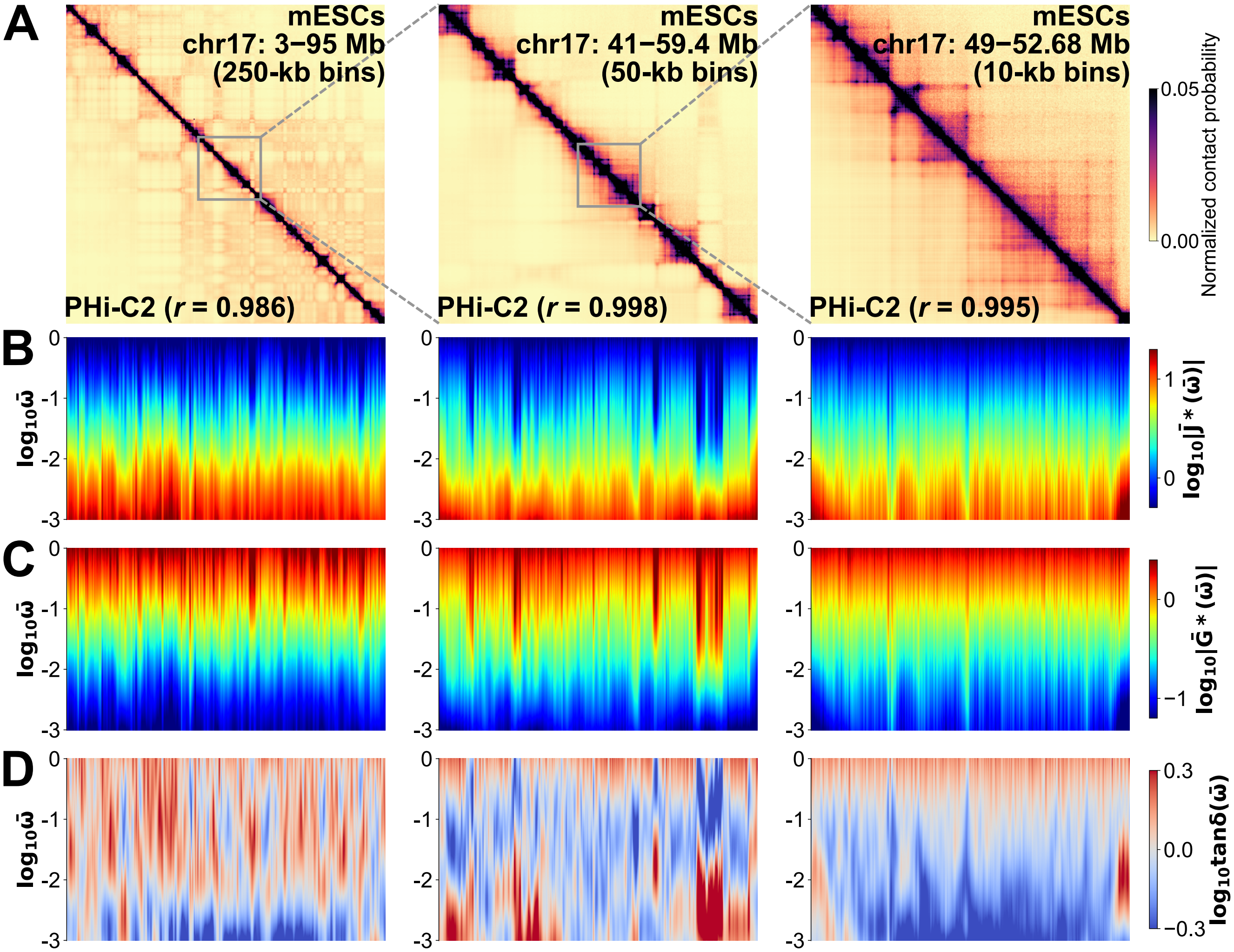

Supplementary Figure S5. Rheology analysis. We used Hi-C data of mESCs [(left) chr17: 3–5 Mb (250-kb bins), (middle) chr17: 41–59.4 Mb (50-kb bins), (right) chr17: 49–52.68 Mb (10-kb bins)]. **(A)** Heatmaps of the contact matrix that consist of the input (upper-right) and the optimal (lower-left) matrices. **(B)** Rheology spectra of the normalized complex compliance $|\bar{J}^*(\bar{\omega})|$ as a measure of the dynamic flexibility of three cases. **(C)** Rheology spectra of the normalized complex modulus $|\bar{G}^*(\bar{\omega})|$ as a measure of the dynamic rigidity of three cases. **(D)** Rheology spectra of the loss tangent $\tan\delta(\bar{\omega})$ as a measure of the liquid-like (> 1) and solid-like (< 1) states of three cases.

References

1. Shinkai, S. *et al.* PHi-C: deciphering Hi-C data into polymer dynamics. *NAR Genom. Bioinform.* **2**, lqaa020, DOI: https://doi.org/10.1093/nargab/lqaa020 (2020).
2. Rao, C. R. & Mitra, S. K. *Generalized Inverse of Matrices and its Applications* (Wiley, New York, 1971).
3. Bonev, B. *et al.* Multiscale 3D genome rewiring during mouse neural development. *Cell* **171**, 557–572.e24, DOI: https://doi.org/10.1016/j.cell.2017.09.043 (2017).
4. Le Treut, G., Képès, F. & Orland, H. A polymer model for the quantitative reconstruction of chromosome architecture from HiC and GAM data. *Biophys. J.* **115**, 2286–2294, DOI: https://doi.org/10.1016/j.bpj.2018.10.032 (2018).
5. Liu, L., Zhang, B. & Hyeon, C. Extracting multi-way chromatin contacts from Hi-C data. *PLoS Comput. Biol.* **17**, e1009669, DOI: https://doi.org/10.1371/journal.pcbi.1009669 (2021).
6. Bianco, S. *et al.* Polymer physics predicts the effects of structural variants on chromatin architecture. *Nat. Genet.* **50**, 662–667, DOI: https://doi.org/10.1038/s41588-018-0098-8 (2018).
7. Zhang, H. *et al.* Chromatin structure dynamics during the mitosis-to-G1 phase transition. *Nature* **576**, 158–162, DOI: https://doi.org/10.1038/s41586-019-1778-y (2019).
8. Shinkai, S., Sugawara, T., Miura, H., Hiratani, I. & Onami, S. Microrheology for Hi-C data reveals the spectrum of the dynamic 3D genome organization. *Biophys. J.* **118**, 2220–2228, DOI: https://doi.org/10.1016/j.bpj.2020.02.020 (2020).
9. Larson, R. G. *The Structure and Rheology of Complex Fluids* (Oxford University Press, 1999).
